# Possible Signatures of Hominin Hybridization from the Early Holocene of Southwest China

**DOI:** 10.1038/srep12408

**Published:** 2015-07-23

**Authors:** Darren Curnoe, Xueping Ji, Paul S. C. Taçon, Ge Yaozheng

**Affiliations:** 1 School of Biological, Earth and Environmental Sciences, University of New South Wales, Sydney, New South Wales, Australia; 2 Yunnan Institute of Cultural Relics and Archaeology, Kunming, Yunnan, China; 3 Place, Evolution and Rock Art Heritage Unit, School of Humanities, Gold Coast Campus, Griffith University, Queensland, Australia; 4 Baise Nationalities Museum, Baise, Guangxi, China

## Abstract

We have previously described hominin remains with numerous archaic traits from two localities (Maludong and Longlin Cave) in Southwest China dating to the Pleistocene-Holocene transition. If correct, this finding has important implications for understanding the late phases of human evolution. Alternative interpretations have suggested these fossils instead fit within the normal range of variation for early modern humans in East Asia. Here we test this proposition, consider the role of size-shape scaling, and more broadly assess the affinities of the Longlin 1 (LL1) cranium by comparing it to modern human and archaic hominin crania. The shape of LL1 is found to be highly unusual, but on balance shows strongest affinities to early modern humans, lacking obvious similarities to early East Asians specifically. We conclude that a scenario of hybridization with archaic hominins best explains the highly unusual morphology of LL1, possibly even occurring as late as the early Holocene.

The hominin fossil records of Europe, West Asia and parts of Africa have long dominated discussions about Late Pleistocene human evolution. Yet, despite its vast size, the East Asian landmass was largely overlooked internationally by palaeoanthropologists for much of the later 20^th^ Century, and was considered to be a backwater with respect to this evolutionary episode^1^,^2^. Over the past decade, however, this has begun to change due to several major discoveries that have highlighted the incompleteness of orthodox views. Important among them has been the discovery of the species *H. floresiensis*^3^,^4^ at Liang Bua Cave, Flores, dating between c74 and c17 ka^5^, with its resemblances to Lower Pleistocene hominins^3^,^4^,^6^.

Yet another enigmatic specimen is the partial mandible recovered from Zhirendong in South China dated >100 ka^7^. While it was assigned to *H. sapiens* by its describers^7^, this designation has been questioned owing to its unusual combination of traits^8^. Moreover, new research on the early Late Pleistocene Xujiayao specimens from North China shows the presence of a previously undocumented morphological complex that has taxonomic implications for the Chinese later Pleistocene hominin record^9^,^10^,^11^. Finally, several recent finds in South China dated between ~130 ka and ~70 ka have led to the suggestion that modern humans (*H. sapiens*) may have appeared much earlier in East Asia than conventional wisdom allows^12^,^13^,^14^, raising the possibility of hitherto unknown populations and variation within the species.

Previously, we have reported the discovery of hominin remains dating to the Pleistocene-Holocene transition at Maludong and Longlin (Laomaocao) Cave in Southwest China that exhibit a large number of similarities to archaic hominins^15^,^16^. In the most complete cranium, early Holocene age (c11.5 ka^15^) Longlin 1 (LL1) (Fig. 1), we documented an extensive number of traits that would be unexpected for a modern human, some of them putative plesiomorphies for Late Pleistocene *Homo*: a finding unseen for any cranium from East Asia during this period and unusual even among Late Pleistocene remains globally^15^,^16^, including Australians^17^. We suggested that two most likely interpretations of the morphology of the Maludong and Longlin Cave fossils were^15^,^16^: 1) they represent the remnants of a precociously early and highly plesiomorphic modern human population that migrated to the region and failed to contribute genetically, or did so in a limited way, to recent people, or 2) they sample a late surviving archaic hominin population.

An alternative interpretation of the affinities of these materials has, however, been offered. Several researchers have questioned our findings and argued that these fossils instead show affinities to either the Late Pleistocene Upper Cave remains from Zhoukoudian^18^ or Late Pleistocene/early Holocene crania from mainland Southeast Asia^19^. A recently described mandible from Tam Pa Ling (TPL2) in nearby Laos has also been described as exhibiting archaic hominin features^20^, although, they are far less numerous than those seen in the Maludong or Longlin remains^15^,^16^. The similarly dated TPL1 cranium is evidently modern in its morphology^19^. Nevertheless, support for the hypothesis that the Maludong and Longlin remains sample a genetically divergent or even late surviving archaic population is growing^21^,^22^,^23^,^24^,^25^. Clearly, if they do show affinities to archaic hominins this would have significant implications for understanding the late phases of human evolution.

Our principal aim here is to test three specific questions about the LL1 specimen: 1) Does its cranial shape closely resemble the Late Pleistocene modern humans from Zhoukoudian Upper Cave (UC101 & UC103) (as proposed by Ref. 18) or Liujiang (LNJG), or even early Holocene mainland Southeast Asians (as proposed by Ref. 19), represented here by the Hang Cho Cave^26^ (HCC) cranium from nearby Northern Vietnam? (2) Alternatively, can the cranial shape of LL1 be accommodated within the range of archaic hominins? (3) Can differences between the morphology of LL1 and modern humans be explained by allometry (i.e. size-scaled shape differences)? To investigate them, bivariate and multivariate methods were applied to craniometric data from LL1. Comparative crania employed were East Asian early modern humans (EMH) represented by UC101, UC103, LNJG and HCC, several European EMH, a putative subspecies of *H. sapiens* (Herto: *H. sapiens idaltu*^27^), several archaic crania and Howells^28^,^29^ large global sample of recent modern humans (RMH) (see Table S1). We examined subsets of the data employing nine and four variables, the latter excluding measurements reconstructed by measuring to the median sagittal plane and doubling the value in LL1 (Table 1).

## Results

### Univariate analysis

First we undertook a univariate examination of the data, which revealed that the value for one raw variable in LL1 (EKB: see Table 1) was outside of (above) the range of 2,524 RMH. The same variable in Herto (HTO) also exceeded the RMH maximum. In Shanidar 1 (SD1) another variable (NPH), and in Shanidar 5 (SD5), two variables (NPH & NLB), were larger than the RMH maximum. In Petralona (PETRA), the value for three variables (ZYB, NPH & EKB) was above the RMH maximum, while the Predmosti 3 (PRD3) cranium exceeded the RMH range for one variable (OBB). Examining size-adjusted variables showed that no measurements in LL1 were outside of RMH range. However, HTO exceeded the global RMH range for one size-adjusted variable (EKB), as did PETRA for a single variable (NPH), while Cro-Magnon 1 (CM1) was below the RMH minimum for one variable (OBH). Finally, values for two variables (NLB & FRC) for the Sima de Los Huesos Cranium 5 (SH5) were above the size-adjusted RMH maximum.

### Principal component analysis

**Figure 2:**
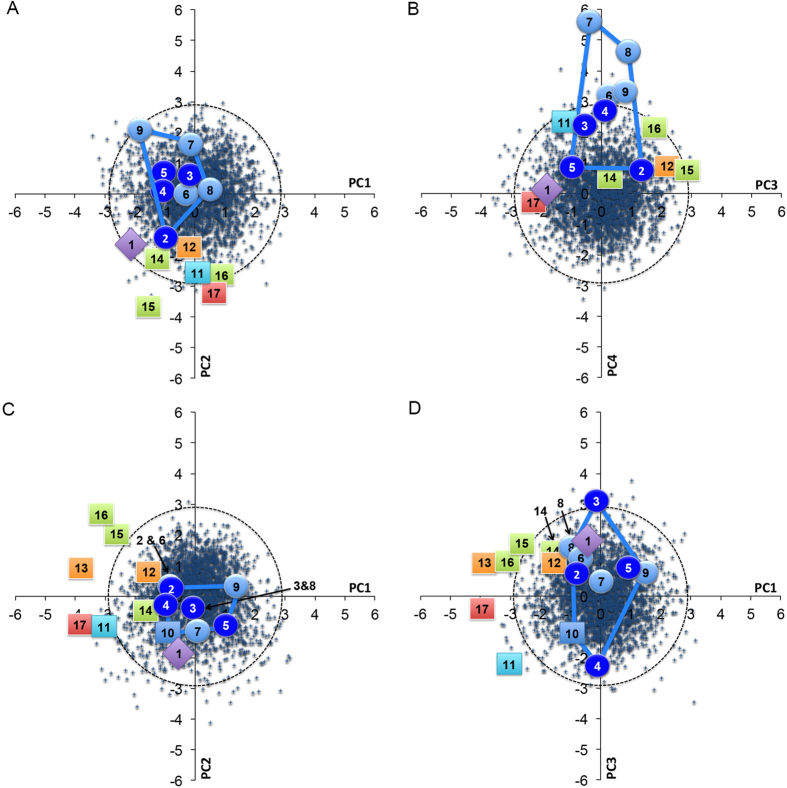
Scatterplots of PCA object scores: (**a**) nine variables, PC1 versus PC2; (**b**) nine variables, PC3 versus PC4; (**c**) four variables, PC1 versus PC2; and (**d**) four variables, PC1 versus PC3 (NB: 95% concentration ellipsis for RMH shown; convex hull for EMH indicated; Key: 1 = LL1, 2 = UC101, 3 = UC103, 4 = LJNG, 5 = HCC, 6 = KEL, 7 = CM1, 8 = PRD3, 9 = PRD4, 10 = OASE 2, 11 = HTO, 12 = SD1, 13 = SD5, 14 = DALI, 15 = SH5, 16 = PETRA & 17 = SANG17; see main text and Table S1 for key to abbreviations).

Next we used principal component analysis (PCA) to examine the phenetic affinities of LL1. In the first PCA, employing nine size-adjusted variables, the first four PCs explained 84.35% of variance (see Table S2). A scatterplot of object scores for PC1 (36.26%) and PC2 (23.74%) showed that most fossils plotted within the 95% concentration ellipsis for RMH, the exceptions being SH5 and Sangiran 17 (SANG17) (Fig. 2A). There was extensive overlap between EMH and archaic crania on PC1, while PC2 separated these groups (Fig. 2A). PC1 mostly sorted crania on the basis of differences in size-adjusted orbit height (OBH), while PC2 contrasted crania on the basis of posterior frontal breadth (STB) (Table S3). Among all of the fossils included, LL1 exhibited the largest negative object score for PC1, plotting outside of the range of all other fossils (Fig. 2A). For PC2, the object score for LL1 was outside of the range of EMH, although, it was close to UC101 as well as the archaic Dali cranium, being virtually identical to SD1 (Fig. 2A).

Although PC3 (14.21%) versus PC4 (10.14%) explained a much lower proportion of variance, a scatterplot of object scores showed interesting contrasts among crania (Fig. 2B). PC3 tended to sort crania largely according to size-adjusted nasal height (NLH) and STB, while PC4 mostly contrasted crania on the basis of size-adjusted bizygomatic breadth (ZYB) (Table S3). On PC3, SD1, SH5 and PETRA were distinguishable from EMH, although, Dali sat within the EMH range (Fig. 2B). On PC4, EMH were characterized by considerable variance, which extended well beyond RMH (positive scores). Thus, this PC highlighted differences between the morphology of non-East Asian EMH and most RMH, with East Asian EMH sitting among RMH (Fig. 2B). PC3 and PC4 clustered LL1 and SANG17 together, both of them exhibiting object scores outside of the range of EMH for both PCs, but within the range of RMH (Fig. 2B).

The second PCA included two additional fossils [Shanidar 5 (SD5) and Oase 2], but used only four size-adjusted variables. Only three PCs were produced and they explained 99.99% of variance (Table S2). A scatterplot of object scores showed that PC1 (53.97%) separated all EMH crania from archaic remains and HTO (Fig. 2C). PC1 was mostly explained by variation in frontal shape: posterior frontal breadth (STB) and frontal length (FRC) (Table S3). PC2 (30.65%) distinguished PETRA, SH5, SD1 and SD5 from EMH, HTO and SANG17 (Fig. 2C). On PC1, LL1 sat within the range of EMH, but on PC2 it was outside of the range of all other fossils (Fig. 2C). Overall, LL1 plotted closest to Oase 2. A scatterplot of object scores for PC1 versus PC3 (15.37%) showed no sorting of groups along the orthogonal axis (Fig. 2D). LL1 sat well within the range of EMH and archaic crania for PC3 (Fig. 2D).

### Neighbor joining analysis

**Figure 3:**
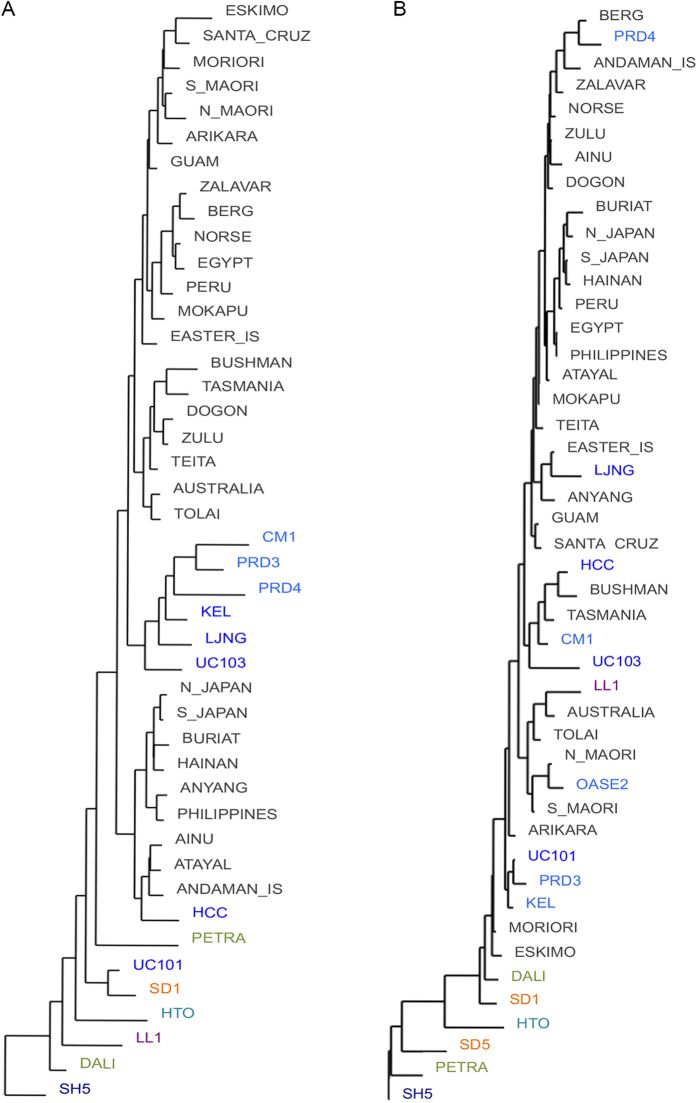
Neighbor-joining trees from cluster analysis using SH5 as an out-group: (**a**) nine variables **a**nd (**b**) four variables (RMH represented by sample means; see main text and Table S1 for key to abbreviations).

The results of neighbor joining (NJ) analysis using RMH sample means, excluding SANG17, and employing SH5 as an outgroup to establish polarities, are provided in Fig. 3. In the first tree, using nine variables, Dali was the most basal branch, followed by LL1, HTO, then a branch containing UC101 and SD1, PETRA, and finally a large branch containing all other EMH and RMH (Fig. 3A). In the NJ tree employing four variables (Fig. 3B), PETRA was the most basal branch, followed by SD5, HTO, SD1 and Dali, and finally a large branch containing all other EMH and RMH (Fig. 3B). In this tree, LL1 was found to reside within the large modern human cluster, within a smaller clade containing recent Australian and Tolai sample means (Fig. 3B).

### Discriminant function analysis

With DFA, even if the independent variable has no relationship to the groups as defined by the dependent variables, we should expect classification predictions to be correct a certain proportion of the time owing to chance alone. The two DFA models we employed exhibited an estimated accuracy of 138% (9 variables) and 158% (4 variables), providing an improvement of 38% and 58%, respectively, over chance alone (see Methods). Moreover, when we examined the classification of RMH in our DFA models we found that 7% (9 variables) and 13% (4 variables) of crania were misclassified, unsurprisingly, emphasizing the greater accuracy of the former. The classification results of DFA showing cross-validated assignments and associated posterior probabilities for all fossils are provided in Table 2.

The first DFA model classified LL1 as non-modern (NM) with a confidence of 58.3%, the second highest group being early modern human (EMH) with a posterior probability of 32.4% (Table 2). Thus, its classification using this model and the groups we employed was ambiguous. UC101 was also classified as NM with a posterior probability of 58.6%, the second highest group being recent modern humans (RMH) with a probability of 41.1%. The classification of UC101 was also, therefore, considered to be ambiguous. All of the other modern human fossils except HCC were, however, firmly classified as EMH with posterior probabilities ranging from 79.9–100% (Table 2). HCC was classified in RMH with a posterior probability of 97.5% (Table 2). Among the other fossils, HTO, SH5 and Dali were correctly classified as NM (Table 2). SD1 and SANG17 were, however, incorrectly classified as RMH, and PETRA as EMH, with high posterior probabilities (Table 2).

The second (4 variable) DFA model classified LL1 as an EMH with a confidence of 74.4%, the second highest group being RMH with a posterior probability of 24.7% (Table 2). Every other modern human fossil was, however, classified as RMH with posterior probabilities ranging from 48.8% (UC103) to 94% (LJNG) (Table 2). This finding underscores the uniqueness of the morphology of LL1 relative to modern humans. Interestingly, UC103, CM1 and PRD3 were the most ambiguous in their classifications according to posterior probabilities, although, the second highest group in all cases was EMH (Table 2). HTO was ambiguously classified as NM (49.6%), its second highest group being EMH (45.1%); a near 50:50 split between them. SH5 and Dali were correctly classified as NM (Table 2). SD5, SH5, PETRA and SANG17 were also assigned to NM, the latter ambiguously according to posterior probabilities (Table 2). SD1 was classified as RMH, while Dali was ambiguously classified as EMH (Table 2).

### Allometry analysis

Size correlated shape (allometric) changes are an important potential source of error in attempts to assess the phenetic affinities of crania using multivariate methods^30^,^31^,^32^,^33^,^34^,^35^. For example, when we compared the average geometric mean of EMH against the average geomean of the RMH sample we found that the difference (65.9 mm v. 63.7 mm) was significant using a 2-way *t-*test (*p*0.03). Moreover, the average geomean for our pooled archaic sample was substantially larger (72.0 mm) than RMH (*p*4.6E-11), and also significantly larger than the EMH average geomean (*p*0.003). LL1, with a geometric mean of 66.7 mm, was found to be intermediate, and not significantly different to, the EMH and RMH averages (*z-*scores 0.23 & 1.07), but significantly smaller than archaic hominins (*z*2.20). Thus, size scaling shape relationships are likely to explain at least some of the differences among crania in our dataset, and although we size-adjusted our data using the geometric mean, this method cannot correct for allometry^36^,^37^.

To examine whether scaling relationships between cranial measurements and size might explain differences between LL1 and modern humans we undertook regression analysis of the logged geometric mean versus logged variables. First, we undertook regression on RMH only to assess whether there was an association between size and each variable using a large sample. As there is disagreement in the literature about which regression model is the most appropriate for allometric studies^38^,^39^, we undertook ordinary least squares and reduced major axis regression (Table S4). All variables were found to be significantly correlated with the geometric mean in RMH (*p* < 0.0001), scaling positively with size (Table S4). Correlation coefficients were weak to moderate (*r*^*2*^ 0.182–0.714) indicating that cranial size expressed as the geometric mean explained between approximately 18% and 71% of variance in these cranial variables (avg. *r*^*2*^ 0.421).

**Figure 4:**
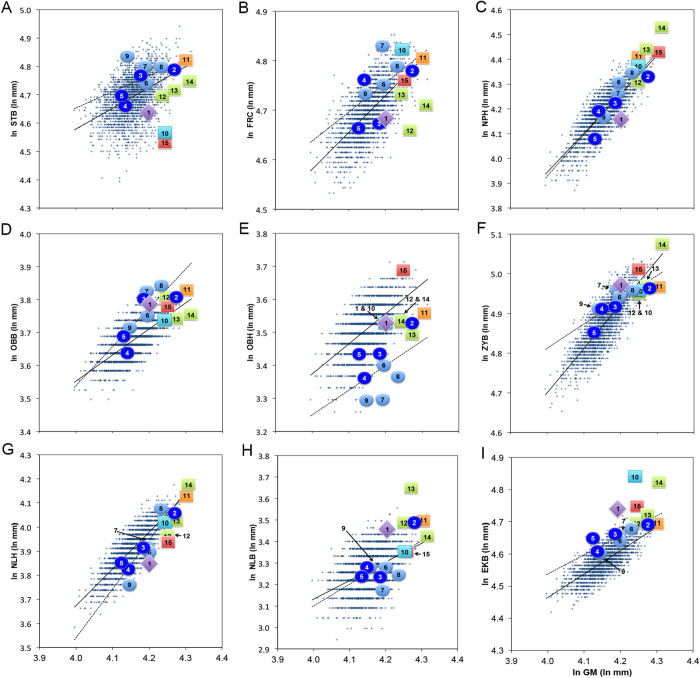
Bivariate plots of logged geomean versus logged craniometric variables: (**a**) STB, (**b**) FRC, (**c**) NPH, (**d**) OBB, (**e**) OBH, (**f**) ZYB, (**g**) NLH, (**h**) NLB, and (**i**) EKB (NB: least squares line of best fit for RMH (unbroken line) and EMH (broken line) shown; Key: 1 = LL1, 2 = UC101, 3 = UC103, 4 = LJNG, 5 = HCC, 6 = KEL, 7 = CM1, 8 = PRD3, 9 = PRD4, 10 = HTO, 11 = SD1, 12 = DALI, 13 = SH5, 14 = PETRA & 15 = SANG17; see main text and Table S1 for key to abbreviations).

Bivariate plots of logged geometric mean versus cranial variables for RMH and fossils are shown in Fig. 4. Visually, it was clear that LL1 departed substantially from the line of best fit for RMH (solid regression line) and/or EMH (broken regression line) for most variables (Fig. 4). It did, however, sit close to the allometric trend line of EMH for OBB, and near the trend line of RMH for OBH (Fig. 4). To test whether departures from the RMH trend line were significant we calculated externally studentized residuals for LL1 and included all EMH crania (Table S5). Externally studentized residuals were found to be significant for LL1 for FRC, NPH, NLH, NLB and EKB (Table S5). This is far in excess of the EMH range of 0–3 significant residuals (Table S5). Moreover, while most (6 out of 8) EMH crania exhibited significant residuals for OBH, it was lacking in LL1 (Table S5).

## Discussion

Returning to the central questions of our study we have found that the cranial shape of LL1 was no closer to East Asian EMH than it was to fossil Europeans. In fact, the results of our multivariate studies clearly indicated that LL1 exhibits shape similarities to both EMH and archaic crania. Importantly, LL1 was sometimes found to be on or towards the edge of the range of EMH and RMH in PCA. It clustered basally with archaic hominins in our 9 variable NJ tree, but with EMH + RMH in the dendrogram produced with 4 variables. It was also classified as non-modern by DFA using our 9 variable model, although, ambiguously in terms of the posterior probability, or alone among modern humans in being classified as EMH (along with Dali) in our 4 variable model.

Our analysis of allometry further confirmed that size correlated shape plays a fundamental role in determining phenetic relationships among hominin crania^30^,^31^,^32^,^33^,^34^,^35^,^36^. Allometry offers the best explanation for the affinities we found between Dali and EMH, the former cranium being small compared to all other archaic remains, and similar in size to EMH. Additionally, the similarities between UC101 and archaic crania, namely Dali and SD1, can also be reconciled in terms of allometry: the Chinese anatomically modern cranium possessing the largest geometric mean of all EMH employed by us. Consistent with these findings is the fact that UC101 failed to exhibit any significant external studentized residuals compared with the allometric line of best fit for RMH.

In contrast, despite its moderate size (geomean 66.7 mm), LL1 was found to show similarities with much larger archaic crania, as well as exhibiting a relatively large number of variables that departed significantly from the size-shape scaling relationship of RMH. Thus, in contrast to UC101, we find that the similarities LL1 shows to archaic crania are much more likely to be of phylogenetic than allometric origin. Therefore, according to our findings, it would simply be incorrect to characterize LL1 as representing a population that is morphologically similar to East Asian EMH^18^,^19^. Additionally, in many instances these features are reminiscent of those seen among Lower Pleistocene remains, and contrary to Ref. 18, most of them have not been observed in EMH regardless of their geological age or geographical provenance^19^,^21^,^22^,^26^,^30^,^31^,^32^,^33^,^34^,^40^,^41^,^42^,^43^,^44^.

When we put the results of our study together it seems clear that the shape of LL1 is highly unusual within the context of variation seen within Late Pleistocene hominin crania. Its distinctive shape is seemingly the consequence of a large number of morphological features seen in archaic crania superimposed on a modern human “gestalt”. Importantly, this combination of archaic and modern features has simply not been found in any archaic cranium. This helps to explain why a definitive classification has eluded us until now^15^, especially within the context of the extensive overlap between archaic and modern crania and the confounding effects of size highlighted in the present and previous studies of Late Pleistocene and RMH crania^30^,^31^,^32^,^33^,^34^,^35^. When we took the influences of allometry (size-shape scaling) into account, however, LL1 remained distinctive, as it did across the results of multiple multivariate techniques using size-adjusted data.

The peculiar mixture of features and overall ambiguous affinities of LL1 are evident also in its non-metric traits (Table 3). This seemingly parallels the situation seen in specimens such as Pestera cu Oase 2 from Romania^45^. The Oase 2 cranium exhibits several hallmarks of modernity, but also possesses a suite of characteristics that distinguish it from Late Pleistocene modern humans^45^. Yet, when considering both metric and non-metric traits, LL1 is even more distinctive than Oase 2, lacks the putative Neanderthal autapomorphies seen in this European specimen, and importantly also dates from the early Holocene rather than mid-Late Pleistocene.

In attempting to explain the morphology of LL1 from a phylogenetic standpoint, two scenarios seem to offer an explanation for the evidence. The first involves evolutionary reversals relative to the presumed ancestral Middle Pleistocene modern human morphology. In our analyses where LL1 was found to be distinctive from most EMH, it was never especially close to the Middle Pleistocene, and presumably plesiomorphic, HTO specimen. Thus, LL1 is unlikely to simply be an EMH that has reacquired a large number of plesiomorphies. At the same time, it was found to be unusual in reference to all of the fossils we employed, making the establishment of polarities all but impossible, and indicating again that the evolutionary reversal scenario is insufficient to explain its morphology and affinities. Moreover, many of the unusual non-metric features of LL1 are rare or absent from EMH regardless of their geological age^19^,^21^,^22^,^26^,^30^,^31^,^32^,^33^,^34^,^40^,^41^,^42^,^43^,^44^, a finding that further undermines such a scenario.

Alternatively, LL1 could descend from a modern population that interbred with one or more archaic groups. This could have resulted in the retention of archaic (plesiomorphic and/or apomorphic) traits as well as the evolution of unique features stemming from novel combinations of genes within the context of modern human gene networks and the disruptive effects of combining genes from divergent gene pools (i.e. hybrid disgenesis). So far, we have been unable to find any trait in LL1 that would be regarded as autapomorphic for *H. neanderthalensis* or any other Late Pleistocene archaic species (Table 3). The absence of archaic remains from mainland East/Southeast Asia reliably dated to <100 ka, excepting the Early *Homo*-like *H. floresiensis*, makes identifying the potential taxon involved in any interbreeding scenario, an important step in identifying hybrids, impossible at present. However, we note that the highly unusual Xujiayao fossils from North China, possibly sampling a novel taxon, could be as young as ~60/70 ka^9^,^10^,^11^. Additionally, DNA evidence suggests that interbreeding with the Neanderthals^46^ and “Denisovans”^47^ by modern humans during the Late Pleistocene could have occurred in East Asia independently of hybridization events in West Asia or Europe^48^, raising the likelihood of multiple pulses of admixture with archaic populations under complex demographic scenarios.

One further clue to the identity of LL1 comes from a recent 3D investigation of the semi-circular canal morphology of a sphenotemporal fragment found along with, and perhaps even belonging to, the LL1 cranium^49^. This work showed that the LL3 temporal labyrinth was indistinguishable from modern humans, being most similar to Holocene humans, and very dissimilar to Neanderthals^49^. If correct, this would indicate an affinity to more recent modern humans, inconsistent with the first (evolutionary reversal) scenario, and raising the possibility of very late occurring hybridization. Indeed, given the extent of the unusual morphological features present in LL1 (Table 3), it could even sample an early Holocene hybrid zone, especially within the context of present understanding of the processes involved in extant non-human primate hybridization^50^ and between Pleistocene modern humans and archaic groups like the Neanderthals and Denisovans^46^,^47^,^48^.

Finally, the morphology of LL1 indicates that just prior to the Neolithic expansions through East Asia human population diversity was unexpectedly large, especially in the region that today includes Southwest China. Moreover, it seems that the evolutionary factors that shaped this diversity were likely to have been highly varied and involved complex demographic scenarios. The distinctiveness of the shape of LL1 forces us to think beyond the usual assumption that hominin crania from the early Holocene must inevitably be fully modern in appearance or ancestry, an observation recently also made for some Late Pleistocene African fossils^51^,^52^.

## Methods

All measurements of LL1 and a cast of Liujiang were taken by DC following the definitions of Howells^28^,^29^. They were blindly checked on multiple occasions across one or more years for accuracy and precision. For example, three estimates of reconstructed ZYB in LL1 – i.e. measured from one side to the median sagittal plane and doubled – taken blindly on separate occasions across two years provided a CV of 0.8%, indicating high precision. Details of the preservation and reconstruction of LL1 can be found in Ref. 15.

The RMH data were downloaded in the form of a Microsoft Excel spreadsheet from *The William W. Howells Craniometric Data Set* (http://web.utk.edu/~auerbach/HOWL.htm) and were sorted it into geographic samples. Fossil data in addition to those collected by us were taken from the literature (Refs. 26,27,53, 54, 55, 56, 57, 58, 59, 60, 61). Unfortunately, the important Minatogawa crania are not complete enough to have been included in our study. Geological dating information was sourced from Refs. 62, 63, 64, 65, 66.

Prior to PCA, NJ-analysis and DFA, all data were converted to size-adjusted (“shape”) variables by dividing the value for each variable by the geometric mean of all measurements for each cranium^36^,^67^. Discriminant function analysis (DFA) using Mahalanobis distances was used to classify crania employing a trichotomous classification (taxonomic free: “recent modern human,” “early modern” and “non-modern”). As very unbalanced matrices may affect the accuracy of the results of this method, we employed a smaller but representative sample of *n*30 randomly selected RMH (rather than n2,524). We used only males to minimize allometric scaling effects, taking the first male cranium each from all 30 of Howells’ geographic samples (see Table S1). To assess the accuracy of each DFA model, we divided the cross-validated accuracy rate by the proportional by chance accuracy rate (summed squared prior probabilities for each group). We considered the results from our models to be accurate if the cross-validated accuracy rate exceeded the chance accuracy rate by at least 25% (heuristic benchmark).

## Additional Information

**How to cite this article**: Cunoe, D. *et al.* Possible Signatures of Hominin Hybridization from the Early Holocene of Southwest China. *Sci. Rep.* **5**, 12408; doi: 10.1038/srep12408 (2015).

## Supplementary Material

Supplementary Information

## Figures and Tables

**Figure 1:**
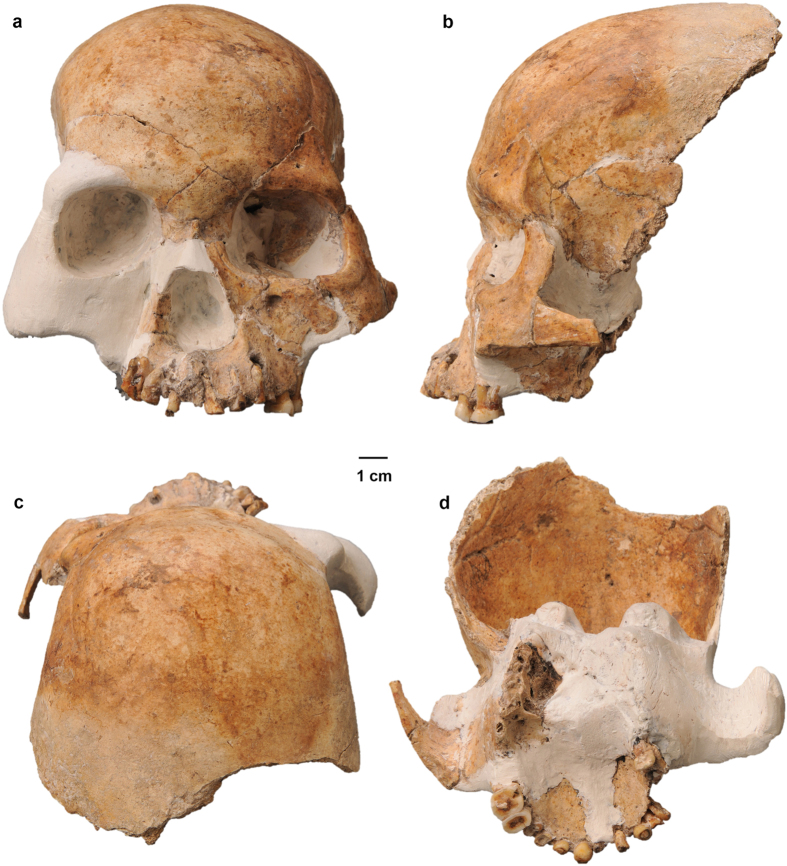
Longlin 1 cranium: (**a**) anterior view, (**b**) left lateral view, (**c**) superior view, and (**d**) inferior view (Photos taken by the authors).

**Table 1:** Craniometric variables employed in the present study and values for Longlin 1 (LL1).

Variable	Abbreviation†	Value for LL1‡
Bistephanic breadth	STB	103
Frontal chord	FRC	108*
Nasion-prosthion height	NPH	64
Orbital breadth	OBB	44
Orbital height	OBH	34
Bizygomatic breadth	ZYB	(144)
Nasal height	NLH	47
Nasal breadth	NLB	(32)
Biorbital breadth	EKB	(114)

^†^Howells (Ref. 28,29) measurement definitions and abbreviations.

^‡^Values in parenthesis reconstructed by measuring to the median sagittal plane and doubling.

^*^Value different from that originally reported in Ref. 15.

**Table 2:** Fossil classifications and associated posterior probabilities for cross-validated results from discrimination function analysis using a simple classification scheme (NB: RMH = recent modern human, EMH = early modern human & NM = non-modern).

	9 Variables†				4 Variables‡			
	Highest group	*p*	2^nd^ highest group	*p*	Highest group	*p*	2^nd^ highest group	*p*
LL1	NM	0.583	EMH	0.324	EMH	0.744	RMH	0.247
UC101	NM	0.586	RMH	0.411	RMH	0.649	EMH	0.311
UC103	EMH	0.962	RMH	0.036	RMH	0.488	EMH	0.480
LJNG	EMH	0.799	RMH	0.189	RMH	0.940	EMH	0.059
HCC	RMH	0.975	EMH	0.025	RMH	0.638	EMH	0.361
KEILOR	EMH	0.985	NM	0.012	RMH	0.709	EMH	0.248
CM1	EMH	1.000	—	—	RMH	0.560	EMH	0.437
PRD3	EMH	0.995	RMH	0.004	RMH	0.535	EMH	0.403
PRD4	EMH	0.998	RMH	0.001	RMH	0.909	EMH	0.090
OASE2	—	—	—	—	RMH	0.692	EMH	0.302
HTO	NM	0.994	EMH	0.006	NM	0.496	RMH	0.451
SD1	RMH	0.930	NM	0.039	RMH	0.634	EMH	0.205
SD5	—	—	—	—	NM	0.964	RMH	0.023
SH5	NM	1.000	—	—	NM	0.845	RMH	0.133
DALI	NM	0.659	EMH	0.385	EMH	0.501	RMH	0.447
PETRA	EMH	0.769	NM	0.224	NM	0.918	RMH	0.077
SANG17	RMH	0.927	EMH	0.072	NM	0.453	EMH	0.350

^†^80.0% of cases were correctly classified.

^‡^66.0% of original grouped cases were correctly classified.

**Table 3:** Unusual (mostly archaic) morphological traits of the LL1 cranium (metrical traits based on comparisons in Ref. 15).

1	Cranial sutures mostly obliterated
2	Thick vault (at *bregma*)
3	Conspicuous supraorbital, with a well developed glabella, and lacking the bipartite form
4	Marked postorbital constriction
5	Broad facial skeleton
6	Mid-face very flat (nasal root and piriform aperture)
7	Nasal bones superiorly very narrow
8	Zygomatic broad, strongly projecting anteriorly (marked alveolar prognathism)
9	Widely flaring zygomatics, angled such that inferior part lies lateral to superior part
10	Anterior masseter attachment marked by broad and deep sulcus
11	Very wide orbits
12	Broad piriform aperture
13	Absence of canine fossa: zygomatic process flat
14	Small zygomatic tubercle
15	Zygomatic tubercle in-line with lateral orbital pillar (anterior view)
16	Interorbital relatively broad
17	Palate broad and shallow, with a prominent anterior shelf
18	Large P^4^ crown
19	Large M^1^ crown
