# Supplementary material for: Possible Signatures of Hominin Hybridization from the Early Holocene of Southwest China
Source: Sci Rep. 2015 Jul 23;5:12408. doi: 10.1038/srep12408 (PMC5378881; doi:10.1038/srep12408)
Supplement: Supplementary Information [file srep12408-s1.doc]

**Supplementary Information**

Curnoe, D., Ji, X., Taçon, P.S.C. & Ge, Y. Possible signatures of hominin hybridization from the early Holocene of Southwest China**.** *Scientific Reports.*

**Table S1**. Fossil specimens and recent modern human (RMH) samples used in present study (see main text Methods for data sources).

| Samples | Classification | Region | Geological Age |
| --- | --- | --- | --- |
| *Individual fossils* (*Abbreviation*) |  |  |  |
| Longlin 1 (LL1) | Unknown | East Asia | Early Holocene |
| Upper Cave 101 (UC101) | *H. sapiens* | East Asia | Late Pleistocene |
| Upper Cave 103 (UC103) | *H. sapiens* | East Asia | Late Pleistocene |
| Liujiang (LJNG) | *H. sapiens* | East Asia | Late Pleistocene |
| Hang Cho Cave (HCC) | *H. sapiens* | East Asia | Early Holocene |
| Keilor (KEL) | *H. sapiens* | Australia | Late Pleistocene |
| Cro Magnon 1 (CM1) | *H. sapiens* | Europe | Late Pleistocene |
| Predmosti 3 (PRD3) | *H. sapiens* | Europe | Late Pleistocene |
| Predmosti 4 (PRD4) | *H. sapiens* | Europe | Late Pleistocene |
| Oase 2 | *H. sapiens* | Europe | Late Pleistocene |
| Herto (HTO) | *H. sapiens* | North East Africa | Middle Pleistocene |
| Shanidar 1 (SD1) | *H. neanderthalensis* | West Asia | Late Pleistocene |
| Shanidar 5 (SD5) | *H. neanderthalensis* | West Asia | Late Pleistocene |
| Sima de los Huesos Cranium 5 (SH5) | *H.* *heidelbergensis* | Europe | Middle Pleistocene |
| Dali | *H.* aff. *heidelbergensis* | East Asia | Middle Pleistocene |
| Petralona (PETRA) | *H.* *heidelbergensis* | Europe | Middle Pleistocene |
| Sangiran 17 (SANG17) | *H. erectus* | South East Asia | Calabrian |
|  |  |  |  |
| *Modern humans* (n2,524) |  |  |  |
| Berg | *H. sapiens* | Europe | Late Holocene |
| Norse | *H. sapiens* | Europe | Late Holocene |
| Zalavar | *H. sapiens* | Europe | Late Holocene |
| Egypt | *H. sapiens* | North Africa | Late Holocene |
| Bushman | *H. sapiens* | Sub-Saharan Africa | Late Holocene |
| Dogon | *H. sapiens* | Sub-Saharan Africa | Late Holocene |
| Teita | *H. sapiens* | Sub-Saharan Africa | Late Holocene |
| Zulu | *H. sapiens* | Sub-Saharan Africa | Late Holocene |
| Australia | *H. sapiens* | Australasia | Late Holocene |
| Tasmania | *H. sapiens* | Australasia | Late Holocene |
| Tolai | *H. sapiens* | Australasia | Late Holocene |
| Easter Island | *H. sapiens* | Pacific | Late Holocene |
| Guam | *H. sapiens* | Pacific | Late Holocene |
| Mokapu | *H. sapiens* | Pacific | Late Holocene |
| Moriori | *H. sapiens* | Pacific | Late Holocene |
| Northern Maori | *H. sapiens* | Pacific | Late Holocene |
| Santa Cruz | *H. sapiens* | Pacific | Late Holocene |
| Southern Maori | *H. sapiens* | Pacific | Late Holocene |
| Ainu | *H. sapiens* | East Asia | Late Holocene |
| Andaman Islands | *H. sapiens* | East Asia | Late Holocene |
| Anyang | *H. sapiens* | East Asia | Late Holocene |
| Atayal | *H. sapiens* | East Asia | Late Holocene |
| Buriat | *H. sapiens* | East Asia | Late Holocene |
| Hainan | *H. sapiens* | East Asia | Late Holocene |
| Northern Japan | *H. sapiens* | East Asia | Late Holocene |
| Philippines | *H. sapiens* | East Asia | Late Holocene |
| Southern Japan | *H. sapiens* | East Asia | Late Holocene |
| Arikara | *H. sapiens* | Americas | Late Holocene |
| Eskimo | *H. sapiens* | Americas | Late Holocene |
| Peru | *H. sapiens* | Americas | Late Holocene |

**Table S2**. Results of principal component analysis (PCA).

| PC | Eigenvalue | %-variance | Cumulative |
| --- | --- | --- | --- |
|  |  |  | %-variance |
| 9 variables |  |  |  |
| 1 | 0.0005 | 36.26 | 36.26 |
| 2 | 0.0004 | 23.74 | 60.00 |
| 3 | 0.0002 | 14.21 | 74.21 |
| 4 | 0.0001 | 10.14 | 84.35 |
| 4 variables |  |  |  |
| 1 | 0.0003 | 53.97 | 53.97 |
| 2 | 0.0002 | 30.65 | 84.62 |
| 3 | 7.72E-05 | 15.37 | 99.99 |

**Table S3**. Variable loadings for PCA (largest correlating variable in bold).

|  | PC1 | PC2 | PC3 | PC4 |
| --- | --- | --- | --- | --- |
| 9 variables |  |  |  |  |
| STB^†^ | -0.10527 | **0.77632** | 0.42295 | -0.13182 |
| FRC | -0.00082 | -0.00736 | -0.23320 | 0.27182 |
| NPH* | 0.44728 | -0.23156 | 0.32653 | 0.23895 |
| OBB | 0.37955 | -0.26469 | 0.34303 | 0.11546 |
| OBH | **-0.71258** | -0.45805 | 0.34136 | -0.20424 |
| ZYB | 0.30704 | -0.05803 | -0.28039 | **-0.83446** |
| NLH | -0.05336 | -0.02878 | **-0.46181** | 0.19055 |
| NLB | -0.18396 | 0.02990 | -0.34949 | 0.15048 |
| EKB | -0.07788 | 0.24225 | -0.10899 | 0.20326 |
| 4 variables |  |  |  |  |
| STB | **0.70448** | 0.45790 | 0.20986 | - |
| FRC | -0.68229 | 0.53023 | -0.05782 | - |
| NPH* | -0.14884 | -0.59651 | 0.60993 | - |
| OBB | 0.12665 | -0.39161 | -0.76197 | - |

*Variable estimated in Dali and SANG17.

^†^Variable estimated in UC103.

**Table S4**. Results of regression of logged geomean versus logged craniometric variables of recent modern human crania.

| Variable | RMAR^†^ |  | OLSR^†^ |  |  |  |  |
| --- | --- | --- | --- | --- | --- | --- | --- |
|  | *Slope* | *Intercept* | *Slope* | *Intercept* | *r^2^* | *t* | *p* |
| STB | 1.651 | -2.263 | 0.704 | 1.722 | 0.182 | 23.71 | <0.0001 |
| FRC | 1.138 | -0.102 | 0.736 | 1.596 | 0.417 | 42.50 | <0.0001 |
| NPH | 1.942 | -3.996 | 1.539 | -2.995 | 0.628 | 65.29 | <0.0001 |
| OBB | 1.168 | -1.247 | 0.804 | 0.289 | 0.473 | 47.60 | <0.0001 |
| OBH | 1.526 | -2.914 | 0.879 | -0.188 | 0.332 | 35.37 | <0.0001 |
| ZYB | 1.361 | -0.859 | 1.149 | 0.027 | 0.714 | 79.40 | <0.0001 |
| NLH | 1.837 | -3.831 | 1.496 | -2.395 | 0.663 | 70.49 | <0.0001 |
| NLB | 2.024 | -5.261 | 0.879 | -0.436 | 0.189 | 24.20 | <0.0001 |
| EKB | 0.995 | 0.386 | 0.436 | 2.740 | 0.193 | 24.48 | <0.0001 |

^†^RMAR=reduced major axis regression; OLSR=ordinary least squares regression.

**Table S5**. Externally studentized residuals.

|  | STB | FRC | NPH | OBB | OBH | ZYB | NLH | NLB | EKB | Total No. |
| --- | --- | --- | --- | --- | --- | --- | --- | --- | --- | --- |
|  |  |  |  |  |  |  |  |  |  | Sign. Res. |
| LL1 | -1.42 | **1.98** | **-2.00** | 1.90 | -0.55 | 1.33 | **-2.89** | **1.98** | **4.16** | 5 |
| UC101 | 0.13 | 1.60 | -0.82 | 0.98 | -1.73 | -1.20 | -0.64 | 1.60 | 1.17 | 0 |
| UC103 | 0.74 | **-0.66** | -0.13 | **2.84** | **-1.97** | 0.42 | -0.51 | -0.66 | 0.11 | 3 |
| LJNG | -0.28 | 0.53 | 0.52 | -0.68 | **-2.55** | 1.66 | -1.29 | 0.53 | **2.09** | 2 |
| HCC | 0.27 | 0.18 | -1.37 | 0.90 | -1.05 | 0.24 | -0.45 | 0.18 | 0.11 | 0 |
| KEIL | 0.34 | -0.07 | 1.05 | 1.40 | **-2.76** | 0.68 | -1.33 | -0.07 | 1.53 | 1 |
| CM1 | 1.16 | -1.51 | 0.33 | **2.96** | **-4.65** | 1.20 | -0.79 | -1.51 | 1.92 | 2 |
| PRD3 | 0.69 | -0.96 | 0.74 | **2.77** | **-4.03** | -0.16 | 1.02 | -0.96 | **2.13** | 3 |
| PRD4 | **2.30** | 0.45 | 0.00 | 1.17 | **-3.89** | 1.42 | **-3.03** | 0.45 | 1.53 | 3 |
